# The World Medical Association: The Voice of Physicians in the Turbulence Between Ethical Rigor and Geopolitical Imperatives

**DOI:** 10.34172/ijhpm.9649

**Published:** 2026-06-13

**Authors:** Meriem Gaddas, Helmi Ben Saad

**Affiliations:** ^1^Research Laboratory LR12SP09 ‘Heart Failure,’ Farhat HACHED University Hospital, Sousse, Tunisia.; ^2^Department of Physiology and Functional Explorations, Farhat HACHED University Hospital, Sousse, Tunisia.; ^3^Faculty of Medicine ‘Ibn el Jazzar’ of Sousse, University of Sousse, Sousse, Tunisia.

**Keywords:** Bioethics, Gaza, Genocide, Humanitarian Crises, Medical Accountability, WMA

## Abstract

This editorial examined the enduring ties between medicine and political power, highlighting how the World Medical Association (WMA) arose in response to recurring ethical crises involving physicians. Created after the Second World War alongside new international institutions, the WMA aimed to protect professional autonomy and defend medical neutrality. Over the decades, it has acted as an ethical counterbalance within global health governance, especially through collaboration with the World Health Organization (WHO). Yet history shows that political pressures have repeatedly compromised medical practice, generating dual-loyalty dilemmas, human rights violations, and scandals that erode public trust. The WMA has sometimes responded firmly—suspending national associations, issuing statements during conflicts, or challenging unethical state policies—though its actual influence remains contested. Internal political divisions have often slowed or limited its actions. Today’s (ie, mid-March 2026) severe degradation of health systems in conflict zones reinforces the need to renew core ethical principles through collective responsibility and vigilance.

 Since antiquity, the role of the physician has extended beyond that of a mere healer.^1^ Owing to their proximity to ruling elites, physicians were often placed at the very center of political affairs, serving as advisors (eg, Galen, physician to Emperor Marcus Aurelius) or even as instruments for consolidating political power.^1^ This interconnection is a contemporary reality, as found in the record of the World Medical Association (WMA)—the paramount global representative body for physicians, encompassing 115 national medical associations (NMAs) and representing over 10 million doctors worldwide.^1^ Established in response to recurring scandals and crises involving physicians, the WMA (website: https://www.wma.net/) has consolidated its role as the primary custodian of the Declaration of Helsinki—the international gold standard for research ethics. Its foundational initiatives, ranging from the post-Nuremberg trial responses to the ethical shifts prompted by the Beecher report (Table 1), represent a long-standing commitment to safeguarding the integrity and normative framework of the profession, and ensuring professional independence.^2^

**Table 1:** Major Ethical Declarations and Statements of the World Medical Association^1,2^

**Date/WMA Declaration **	**Historical Context, Triggering Events and Specific ** **Evidence of Misconduct**	**Paradigm Shift (Analytical Value)**
1948 – Declaration of Geneva	Historical context: Post-Second World War/Nuremberg "Doctors’ Trial" (1946–1947): Systematic collapse of medical ethics under totalitarianism. Triggering events: 23 high-ranking physicians (eg, Karl Brandt) indicted for prioritizing Rassenhygiene (racial hygiene) and "State interest" over the Hippocratic Oath. Specific evidence: Direct involvement in forced euthanasia and eugenic programs, rationalizing clinical atrocities as "state necessity.”	Core conceptual shift: Transition from "state servitude" to "professional autonomy.” Establishes the physician’s primary loyalty to the patient as an absolute principle superseding national law. Institutional impact: Explicit abolition of the "superior orders" defense. It codifies a "duty of disobedience," against state mandates that violate human rights.
1956 (Revision 2004) – Armed Conflict	Historical context: Cold war and de-colonial wars (eg, Algerian War): Systemic pressure on military physicians to prioritize "military victory" over neutrality. Triggering events: Systematic repurposing of medical professionals as "tactical assets" in counter-insurgency operations. Specific evidence: Documented cases of physicians enhancing "interrogation efficiency" and subordinating clinical duties to "military necessity.”	Core conceptual shift: Transition from "military subordination" to “ethical immutability.” Asserts that medical ethics are non-negotiable, even in war. Institutional impact: Formal codification of medical neutrality. Prohibits prioritization tactical goals over clinical obligations, stripping military hierarchy of power over medical priorities.
1964 – Declaration of Helsinki	Historical context: Post-Nuremberg Research Abuses (1950s–1970s): Prioritization of scientific progress over individual rights in democratic states. Triggering events: Henry Beecher’s 1966 report exposing 22 unethical studies. Specific evidence: Systematic experimentation on vulnerable populations (Tuskegee Syphilis Study, Willowbrook hepatitis study) without informed consent.	Core conceptual shift: Transition from "scientific utilitarianism" to "subject sovereignty.” Primacy of the subject's well-being over the interests of science or society. Institutional impact: Institutionalization of independent ethical oversight (Ethics Committees/IRBs), stripping physician-researchers of sole discretionary power.
1975 – Declaration of Tokyo	Historical context: 1960s–1980s Dictatorships and the "double loyalty" crisis: Systematic co-option of physicians by military juntas and authoritarian regimes. Triggering events: Global scandals exposing physicians prioritizing loyalty to repressive regimes (USSR). Specific evidence: Physicians calibrating pain during torture (Chile, Brazil) and the "pathologization" of political dissent (USSR "Sluggish Schizophrenia").	Core conceptual shift: Transition from "state auxiliary" to "moral counter-power.” Defines the physician as an active barrier against state violence. Institutional impact: Absolute prohibition of any participation in torture. Establishes medical ethics as superior to sovereign orders, legitimizing institutionalized civil disobedience.
1981 (Revision 2005) – Declaration of Lisbon	Historical context: 1970s Rise of patient rights movements: critique of medical paternalism and state control over the body. Triggering events: Social movements challenging the absolute power of physicians and demanding therapeutic choice. Specific evidence: Non-consensual psychiatric treatments and denial of medical information in state-run healthcare systems.	Core conceptual shift: Transition from "medical paternalism" to "individual self-determination.” Shifts power from the institution (hospital/state) to the individual. Institutional impact: Formalization of the right to choice and information, mandating that clinical legitimacy rests solely on the patient’s explicit consent.
1987 – Declaration of Madrid	Historical context (Rise of managed care and bureaucratic medicine): Subordination of medical practice to administrative, state, and financial management. Triggering events: Institutional encroachment by third-party payers (governments/insurers) on the clinical autonomy. Specific evidence: Administrative interference where cost-containment measures override therapeutic necessity and independent judgment.	Core conceptual shift: Transition from "administrative subordination" to "clinical autonomy as a patient right.” Professional independence is framed as a safeguard for patient safety rather than a corporate privilege. Institutional impact: Sanctuarization of independent medical judgment. Delegitimizes interference by non-medical tiers (States, insurers) in therapeutic decisions.
1981 (Reaffirmation 2008) – Death Penalty	Historical context: Judicial instrumentalization: State-led co-option of medical knowledge to "humanize" capital punishment. Triggering events: Institutionalization of lethal injection as a primary execution method, creating a functional dependency on medical skills. Specific evidence: Physicians participating in lethal injection protocols to ensure a "painless" procedure and certifying death.	Core conceptual shift: Transition from "judicial auxiliary" to "ethical demarcation.” Establishes a definitive "firewall" between the healing arts and state-sanctioned killing. Institutional impact: Absolute prohibition of medical participation in executions, detaching medical authority from judicial and political power.
1988 – Declaration of Edinburgh	Historical context: Global crisis in medical education: Disconnect between hospital-centric training and the socio-ethical needs of the population. Triggering events: Critique of the "ivory tower" model of medical schools producing technicians rather than socially responsible physicians. Specific evidence: Systemic neglect of ethics and communication curricula, leading to the "dehumanization" of care.	Core conceptual shift: Transition from "techno-scientific specialization" to "social responsibility.” Re-centers medical training on the actual health needs of the community. Institutional impact: Global reform of medical curricula, integrating ethics and social sciences to produce physicians capable of acting as socially responsible agents.

Abbreviations: USSR, Union of Soviet Socialist Republics; WMA, World Medical Association; IRBs, institutional review boards. Note: This table offers a critical appraisal of WMA norms, conceptualizing their evolution as a defensive response to state co-optation rather than a linear bioethical trajectory. Based on the principle of epistemological rupture, the selected texts delineate a shift from an “ethics of obedience” to a paradigm of disseminative ethics and professional resistance.

 Indeed, its positions on certain issues have neither achieved unanimity among its members nor met the expectations of the international community regarding its commitment to neutrality. Table 1 outlines these foundational paradigms and the organization’s historical strengths.^1,2^ Yet, despite its declarations and public positions, the course of history demonstrates that this ultimate goal can never be fully guaranteed.^1^ Instead, it requires continuous reinforcement, with the WMA often engaged in a delicate balancing act aimed at preserving medical autonomy from political spheres and geopolitical interests.^1^

 Nevertheless, the history of the WMA inevitably reminds us of the enduring intersection between medicine and politics.^1^ It is noteworthy that the WMA was founded almost simultaneously with the creation of major United Nations (UN) institutions (the UN in 1945 and the world health organization (WHO) in 1948). At that time, physicians sought to distinguish themselves by establishing an independent international body through which they could defend the values of their profession.^2^ Paradoxically, the WMA initially chose to locate its headquarters in New York —close to that of the UN—to visibly affirm its cooperation with international institutions, before later relocating to France. In practice, the collaboration between the WMA and the UN, primarily through the WHO, has been close.^2^ Schematically, the WMA positions itself as the “*ethical arm*” of global medicine, whereas the WHO assumes the role of its “*technical arm*.”

 Moving beyond this functional synergy, the WMA describes itself as having, over time, sought to distance itself from geopolitical interests by defending its status as an autonomous moral authority within international debates (eg, human rights, opposition to torture, and medical ethics in times of war).^1^ Its stances against prevailing international politics have oscillated across three dimensions as: (*i*) an ally of UN principles (human rights, global health); (*ii*) a moral counterweight when the politicization of crises endangered global health (eg, Taiwan, COVID-19); and (*iii*) a humanitarian sentinel in armed conflicts.In this editorial, contemporary crises—notably in Ukraine, and Gaza—were utilized as stress-test case studies to illustrate broader structural tensions between universal principles and geopolitical realities. We acknowledge that while reports from non-governmental organizations are instrumental for the WMA’s early ethical monitoring and “watchdog” function, they constitute a specific category of preliminary evidence that must be interpreted alongside legal findings and institutional reports from multilateral bodies.

 An examination of the WMA’s interventions over the past fifty years reveals a structural tension between the universality of its principles and the reality of geopolitical power dynamics (Table 2). This analysis highlights an asymmetry in institutional responsiveness—at times characterized by critics as a form of “selective silence”—best illustrated by the contrast between the unprecedented resolve elicited by the conflict in Ukraine and the “strategic prudence” observed in other theaters such as Syria, Yemen, or Gaza.^3,4^ While the former led to immediate institutional sanctions, the latter cases demonstrate a preference for procedural neutrality over humanitarian urgency.^4^

**Table 2:** Analytical Matrix of the World Medical Association Political Stances: Impact, Uptake, and Critical Framework

**Crisis, Date and Nature of Evidence/Allegations**	**WMA Response to the Crisis (Action and Positioning)**	**International Impact (Nature, Degree and Appropriation)**	**Institutional and Analytical Controversies**
**Stances Regarding Member Associations and Governments**			
[Confirmed historical record/Judicial findings] Apartheid (South Africa) 1970–1985^5^: Systemic racism Steve Biko case (1977): Medical complicity in torture (Drs. Lang and Tucker); MASA subordination to State security.	Fluctuating sanctions: Initial suspension* (1976), controversial readmission (1981), and final expulsion of MASA (1985).	Nature: Institutional and normative. Degree: High. Appropriation: Prompted WHO to break official ties with WMA (1982–1987), forcing a shift toward human rights-centered ethics.	Temporal disjunction: 8-year delay between documented violations and expulsion. 1981: Readmission analyzed as "politicized leniency" influenced by major Western NMAs.
[Confirmed historical record] USSR punitive psychiatry 1970–1989^6^: Systematic misuse of psychiatry for political neutralization ("sluggish schizophrenia" diagnosis).	Institutional exclusion: Forced withdrawal of the Soviet medical association (1983). Readmission (1989) conditioned on structural reforms.	Nature: Diplomatic and policy. Degree: High. Appropriation: Direct precursor to the UN principles for the protection of persons with mental illness (1991).	Geopolitical correlation: Contrast between rapid resolve against the Eastern Bloc and the "strategic prudence" observed in other concurrent crises.
[Reported allegations/independent tribunal findings] China - Uyghurs and Organ Harvesting 2020–Present: Reported forced sterilizations; allegations of systematic organ harvesting from prisoners of conscience (Xinjiang).	Declaratory firmness: Resolutions labeling reported acts as "crimes against humanity.” Maintaining CMA membership for "critical engagement.”	Nature: Discursive and symbolic. Degree: Moderate. Appropriation: Utilized by Western associations (BMA) to pressure national governments; rejected by the CMA.	Sanctioning asymmetry: Disparity between verbal condemnation and the lack of membership sanctions, suggesting economic realpolitik constraints on ethical enforcement.
**Stances in Conflict With Multilateral Bodies (UN/WHO)**			
[Diplomatic and policy context] Taiwan - WHO exclusion 2005–Present: Exclusion of 23 million people from global health networks due to UN resolution 2758.	Normative advocacy: Repeated resolutions (2021) supporting Taiwan’s observer status at the WHA, prioritizing health over UN diplomacy.	Nature: Policy and identitarian. Degree: Moderate. Appropriation: Strengthened WMA’s image as a "normative sovereign" capable of defying UN consensus.	Inconsistency in activism: The high level of resolve regarding Taiwan contrasts with the institutional restraint observed in other sensitive Asian human rights dossiers.
[Documented health crisis] Ebola (West Africa) 2014–2016: Systemic infrastructure collapse; bioethical tensions regarding experimental treatments (ZMapp); global mobilization triggered by North-bound health security risks.	Protective advocacy: Normative pressure on UN/WHO to prioritize frontline staff safety; affirmation of infrastructure integrity as an ethical prerequisite; call for systemic health system strengthening.	Nature: Normative and discursive. Degree: Moderate. Appropriation: Instrumentalized by local NMAs to demand PPE and security guarantees from state and international bodies; "ethical conscience" framing.	Distributive justice asymmetry: Reactive urgency (threat to Western borders vs. local humanitarian need); "two-tier ethics" (priority access for repatriated staff); critique of a West-centric ethics failing to address structural under-investment.
[Documented global health crisis] COVID-19 pandemic 2020–2022: Vaccine nationalism; disparities in global distribution; state pressure on clinical data integrity.	Ethical guardianship: Condemnation of vaccine hoarding; promotion of inclusive governance; defense of medical whistleblowers.	Nature: Normative and discursive. Degree: Contested. Appropriation: Utilized by NMAs (eg, USA, Brazil) to defend professional autonomy against political interference.	Declaratory vs. executive gap: Analysis of a role limited to moral guardianship without influence over global IP (TRIPS waiver) or logistics.
**Comparative Analysis of Institutional Resolve in Armed Conflicts**			
[Documented violations/ongoing legal proceedings] Ukraine 2022–Present: Targeted destruction of healthcare infrastructure; 2022 Russian invasion.	Maximalist mobilization: Categorical condemnation; formal designation of an "aggressor"; suspension of the Russian medical association (2022).^4^	Nature: Policy and institutional. Degree: High. Appropriation: Closely aligned with Western multilateral sanctions, legitimizing the professional isolation of the RMA.	Institutional baseline: Establishes a high-responsiveness precedent ("Benchmark"), highlighting by contrast the "neutrality" adopted in other theaters.
[Ongoing investigations/reported allegations] Gaza 2023–2024^4^: Reports of systemic healthcare collapse; reported 1500+ workers killed; unprecedented destruction of infrastructure.	Symmetrical neutrality: Generic calls for ceasefire and hospital protection. Non-attribution policy regarding the parties responsible for destruction.	Nature: Discursive and symbolic. Degree: Low/contested. Appropriation: Challenged by regional bodies (SAMS) and international (NGOs) for lack of an investigative mandate.	Asymmetric responsiveness: Shift from "aggressor-victim" (Ukraine) to "symmetrical/all parties" rhetoric, perceived as a devaluation of Global South clinical crises.
[Judicial Findings/Confirmed historical record] Abu Ghraib (Iraq) 2003: Systematic torture; medical personnel documented falsifying death certificates and calibrating interrogation pain.	Doctrinaire Strengthening: Updated the Declaration of Tokyo (2004); reaffirmed the absolute prohibition of physician participation in torture.	Nature: Ethical and Normative. Degree: High (Theoretical)/Low (Accountability). Appropriation: Integrated into academic bioethics; failed to yield institutional sanctions.	Accountability discontinuity: Absence of proceedings against the AMA, suggesting ethics are subordinate to the interests of dominant powers.
[Reported violations/documented war crimes] Syria/Yemen 2011–Present: Systematic "double tap" strikes on hospitals; militarization of healthcare access.	Humanitarian minimalism: Late and intermittent responses; reliance on generic neutrality language; minimal perpetrator naming.	Nature: Discursive and marginal. Degree: Low. Appropriation: Minimal impact on belligerent conduct; generated a sense of institutional abandonment among local practitioners.	Geographies of concern: Evidence of a hierarchy where institutional resolve is indexed to geopolitical and geographical proximity to Western interests.
**Stance in Defense of Detained Physicians**			
[Documented legal proceedings/human rights advocacy] Turkey - Dr. Serdar Küni 2016–Present: Criminalization of care for treating patients without state authorization in conflict zones.	Protective advocacy: Direct diplomatic pressure; coordination with CPME, FIDH and OMCT to internationalize the judicial defense.	Nature: Judicial and protective. Degree: Moderate. Appropriation: Created a "symbolic shield" preventing the practitioner's disappearance; used by the TTB to monitor trial fairness.	The structural glass ceiling: Demonstrates the limits of moral authority against repressive state sovereignty; inability to provide direct legal or coercive protection.

Abbreviations: AMA, American Medical Association; BMA, British Medical Association; CMA, Chinese Medical Association; CPME, Standing Committee of European Doctors (Comité Permanent des Médecins Européens); FIDH, International Federation for Human Rights (Fédération Internationale pour les Droits de l’Homme); IP, Intellectual Property; MASA, Medical Association of South Africa; NGO, Non-Governmental Organization; NMA, National Medical Association; OMCT, World Organization Against Torture; PPE, Personal Protective Equipment; RMA, Russian Medical Association; SAMS, Syrian American Medical Society; TRIPS, Trade-Related Aspects of Intellectual Property Rights; TTB, Turkish Medical Association (Türk Tabipleri Birliği); UN, United Nations; USA, United States of America; USSR, Union of Soviet Socialist Republics; WHA, World Health Assembly; WHO, World Health Organization; WMA, World Medical Association. Remarks: (1) The crises selected in this table represent inflection points where the autonomy of the medical profession was confronted by systemic state pressure. The impact is analyzed across three dimensions: *Political* (sanctions and concrete actions), *Normative* (the establishment of ethical standards), and *Discursive* (influence on international debate). (2) * It should be clarified that in 1975, following international condemnation of its failure to protect physicians exposing detainee abuse, the MASA withdrew from the WMA of its own accord rather than being formally suspended. The 1976 date often cited refers to the formalization of this absence in subsequent assemblies. (3) Note on Evidentiary Framework: In accordance with methodological standards, this table distinguishes between “Confirmed Historical Records/Judicial Findings” (cases settled by international courts or consensus) and “Reported Allegations/Ongoing Investigations” (contemporary crises where legal proceedings or independent findings are currently being evaluated).

 Far from constituting absolute moral imperatives, the WMA’s stances thus emerge as the product of complex political and financial compromises, representing a deviation from its established normative benchmarks, and often mirroring the diplomatic cleavages observed within the UN. By indexing its positions to the Western agenda, the organization risks being perceived as an instrument of soft power, thereby deepening the rift with the “Global South” and ultimately questioning its claim to truly universal moral authority.^1^ It must also be acknowledged that the WMA has not only confronted external political pressures but has likewise been compelled to navigate internal dissent, often shaped by the political affiliations of its member associations.^1^ Indeed, its positions on certain issues have neither achieved unanimity among its members nor met the expectations of the international community (eg, medical association of South Africa withdrawal in 1975, Table 2).

 At present (ie, mid-March 2026), the medical profession remains confronted by significant deontological challenges, characterized by documented allegations involving certain practitioners in unethical acts. These range from the incitement to target hospital infrastructure^7^ to participation in acts of torture^8^ and the failure to report systemic abuses.^9^ In this context, the healthcare crisis in Gaza exemplifies the severity of these challenges.^3,10^ The systematic reliance on the principle of the “presumption of innocence” is increasingly scrutinized in light of the accumulating findings from international bodies,^10^ forensic expertise, and reports from UN special rapporteurs regarding conduct that may potentially constitute genocidal acts.^10^ The fact that several of these allegations are currently the subject of proceedings before the international court of justice and other international criminal jurisdictions underscores the structural dimension of this crisis.^8^ Consequently, the absence of a formal stance concerning the documented destruction of the healthcare system raises critical questions concerning the consistency of the organization’s normative benchmarks.^10^ In this regard, it is imperative to distinguish between “*professional neutrality*,” which mandates treating every human being without distinction, and “*institutional neutrality*,” which, in the face of systemic crimes, risks being interpreted as institutional inaction.^3,10^ This distinction is rooted in the concept of “dual loyalty” championed by the WMA,^1^ which asserts that absolute fidelity to the patient’s health and integrity must systematically supersede any allegiance to the State, the administration, or the military.^1^ This principle finds its normative cornerstone in the declaration of Tokyo (Table 1), which strictly prohibits any participation in torture or interrogations, thereby establishing medical civil disobedience as a duty whenever the political order violates fundamental rights.^1^

 To be effective, such ethical resistance requires a sanctualized medical neutrality, guaranteeing the inalienable right to treat even “the enemy” without fear of the medical act being criminalized by authorities.^1^ It also necessitates unwavering professional independence, ensuring that NMAs do not become mere conduits of state power.^1^ To achieve this, the WMA must provide the necessary protection for the practitioners it represents.^1^ However, the emblematic case of the Turkish physician “Dr. Serdar Küni” illustrates the intrinsic limitations of the WMA’s actual influence.^11^ While the organization succeeds in internationalizing the defense of persecuted practitioners, it systematically encounters a structural glass ceiling. Lacking coercive power, its condemnation reveals the inherent limitations of a purely declaratory authority, whose influence is constrained when it comes to concretely protecting physicians from the arbitrary power of authoritarian regimes.^11^ Likewise, it must be noted that the WMA’s independence was tested by major structural tensions throughout 2024 and early 2025, underscoring the ongoing friction between ethical universalism and geopolitical realities. By ratifying the resolution “defending the ethical framework of healthcare,” the WMA officially recognized that scientific integrity and human dignity are now targets of political and ideological pressure.^12^ This tension was particularly evident in the North-South rift that emerged during the 2024 revision of the Declaration of Helsinki regarding post-trial access, where the imperatives of the developed world clashed with the equity-seeking agendas of emerging nations.^13^ Such shifts reveal the outsized role played by dominant NMAs, whose financial leverage and political reach largely dictate the organization’s direction.^13^ However, this traditional Euro-American hegemony is increasingly challenged by the strategic coordination of the BRICS countries (ie, five countries: Brazil, Russia, India, China, and South Africa). This emerging Global South bloc seeks to function as an institutional counter-power, aiming to rebalance global discourse and promote a medical ethics framework that is more representative of global interests.^14^

 Furthermore, the WMA’s institutional architecture cannot be detached from the colonial and post-colonial legacies that shaped the post-Second World War global health order.^15^ The formation of global governance bodies, including the WHO and WMA, occurred within a Western-centric framework that inherently defined the standards of moral leadership and ethical normativity according to Northern priorities.^15^ This historical legacy, as emphasized by the decolonizing global health movement, continues to foster knowledge hierarchies and epistemic injustice.^15^ As emphasized by the decolonizing global health movement, these structures often result in a form of epistemicide, where the suffering and narratives of the Global South are marginalized or subjected to a higher threshold of ‘scientific validation’ before triggering institutional resolve.^15^

 As documented,^16^ peer-reviewed journals and medical bodies act as influential mediators in this structural discourse, frequently amplifying Western-aligned crises while relegating others to a state of “strategic prudence.” This ecosystem of global health communication, marked by narrative asymmetry, underscores that contemporary demands for greater ethical consistency and accountability—notably regarding the crises in Gaza, Syria, or Yemen—are not merely political critiques; they represent a fundamental call to decolonize medical ethics.^16^ Ensuring that the WMA’s moral authority transcends these underlying colonial hierarchies and epistemic biases is essential for preserving its claim to a truly universal and equitable mandate.

 This institutional friction also has profound implications for the UN’s 2030 Agenda. While the WHO’s contemporary rhetoric emphasizes *“Health as a human right”* the WMA’s selective resolve reveals a persistent gap between global policy and ethical practice.^1^ Systematic attacks on healthcare infrastructure, when met with institutional silence, directly jeopardize the targets of sustainable development goal (SDG) 3 (ie, good health and well-being) and exacerbate the disparities addressed by SDG 10 (ie, reduced inequalities). Furthermore, the WMA’s credibility as a normative guardian is essential to SDG 16 (ie, peace, justice, and strong institutions); inconsistent accountability regarding systemic abuses undermines the institutional strength required to protect the sanctity of care in conflict zones.^1^ Failing to align ethical output with these global frameworks risks transforming universal slogans into hollow rhetoric, further deepening the divide between the administrative elite and practitioners on the frontlines.^1^

 To restore its moral authority, the WMA must move beyond a purely declaratory role toward more robust governance. This requires the development of objective thresholds for assessing violations of medical neutrality, ensuring that institutional resolve is triggered by the severity of the crisis rather than its geopolitical proximity. Central to this effort should be the establishment of an independent observatory to monitor attacks on healthcare, alongside protected channels for frontline whistleblowers and support mechanisms for NMAs facing state repression. Internal integrity could be further reinforced through stricter conflict-of-interest disclosures and a deliberate diversification of leadership to foster equitable representation of the Global South.Fulfilling the need for a truly independent global medical authority depends on these structural reforms. Ultimately, periodic external audits and enhanced digital transparency would ensure that the WMA remains a resilient guardian of international humanitarian law, transforming the promise to ‘leave no one behind’ into a functional reality rather than a symbolic aspiration.

 In conclusion, the history of the WMA underscores a persistent tension between the ideals of medical ethics and the harsh realities of the field. By enshrining the right to health as a universal imperative, the organization frames the act of providing care as a sovereign ethical and political commitment. In the face of systemic abuses, market-driven pressures, and geopolitical divides, this commitment emerges as the ultimate bulwark for medical autonomy and human dignity against the interests of the State.

**Take-Home Message**

The WMA embodies the enduring tension between medicine and politics; while it was founded to defend medical ethics and physician independence, history shows that this mission is constantly challenged by political pressures, internal divisions, and global crises. To preserve its moral authority and maintain public trust, the WMA and its member associations must consistently uphold the primacy of medical ethics and speak out against violations, especially in times of conflict and repression.

## Acknowledgments

 The authors would like to thank (*i*) Pr. Leslie London (School of Public Health, University of Cape Town, South Africa) for providing valuable historical insights and for his constructive suggestions, which significantly improved the final version of this manuscript, and (*ii*) the assistant editor and the reviewers for their insightful, constructive, and thorough evaluation of our manuscript. The feedback provided has been instrumental in enhancing the conceptual clarity, analytical depth, and overall rigor of our work.

## Disclosure of artificial intelligence (AI) use

 The authors would like to reveal that in order to improve the consistency and clarity of the manuscript authoring, artificial intelligence tool (ie, Google Studio AI) was used. Without changing the scientific substance or creating any new material, the tool was used solely for language improvement, making sure the text was understandable and cohesive.

## Ethical issues

 Not applicable.

## Conflicts of interest

 Author declares that he has no conflicts of interest.
